# Characterization of Small Genetic Variants in Breast Cancer Cell Line Under Tamoxifen Therapy: Alterations of Small Genetic Variants Under Tamoxifen Therapy

**DOI:** 10.31661/gmj.v12i.2598

**Published:** 2023-06-26

**Authors:** Mahnaz Nezamivand-Chegini, Hamed Kharrati-Koopaee, Seyed Taghi Heydari, Hasan Giahi, Fatemeh Sabahi, Ali Dehshahri, Kamran Bagheri Lankarani

**Affiliations:** ^1^ Institute of Biotechnology, Shiraz University, Shiraz, Iran; ^2^ Health Policy Research Center, Institute of Heath, Shiraz University of Medical Sciences, Shiraz, Iran; ^3^ Department of Plant Protection, College of Agriculture, Shiraz University, Shiraz, Iran; ^4^ Department of Pharmaceutical Biotechnology, Shiraz University of Medical Science, Shiraz, Iran

**Keywords:** Tamoxifen, Breast Neoplasms, RNA-seq

## Abstract

**Background:**

Tamoxifen (TAM) is an effective hormone therapy in order to reduce the risk of cancer recurrence. According to the available findings, TAM contributes to the alterations of genetic variants background and may have role in the effectiveness of treatments via alteration of the genetic variants. The effects of TAM on genomic features were investigated in current study through discovering genetic variants and finding the answer of the following question: “Is there any association between the alterations of genetic variants under TAM consumption and an effective treatment process?”

**Materials and Methods:**

Whole-transcriptome (RNA-seq) dataset of four investigations including 10 TAM-treated samples and 9 untreated samples as the control groups were derived from European Bioinformatics Institute (EBI). Using the process of variants calling, the differential genetic variants between and gene ontology enrichment analysis were detected through CLC Genomics Workbench (12).

**Results:**

In current study, almost 5.8 million genetic variants were reported. The outcomes of chi-square test showed that distributions of genetic variants between control and treated samples were significant (P0.05). The genetic variants comparison between the control and TAM-treated samples indicated that there were 67 differential genetic variants. Gene ontology enrichment analysis indicated that differential genetic variants were associated with several tumor suppressors and oncogenes such as IL6ST, GEN1, FNTA. HSPA5, NSMCE2, and DDX11.

**Conclusion:**

Most of the candidate genes with differential genetic variants had dual roles as oncogenes or tumor suppressors. Therefore, it can be claimed that TAM has no significant role in an effective treatment through alteration of the genetic variants. In other words, it cannot be concluded that the TAM therapy-resulted alterations of genetic variants have positive or negative roles in the treatment process.

## Introduction

Cancer is one of the most important causes of mortality in the world and breast cancer is the second most common disease among women [1].

Hormonal therapy is a medical strategy for breast cancer treatment [2]. Tamoxifen (TAM) is considered as the main non-steroidal drug in the breast cancer treatment for postmenopausal women [3], which inhibits the estrogen activity through binding to the estrogen receptor competitively [4].

There are several investigations carried out with the purpose of illustrating the hormonal therapy effects that provide a better understanding of the drug response mechanism and select an effective strategy for the therapeutic period [5][6][7].

The appropriate drug response is a complex interdependent procedure that is highly dependent upon several factors, including the genetic variants background, lifestyle, climate, smoking, and alcohol consumption [8].

Genetic variants refer to the genetic differences between individuals of a population [9]. DNA is a vulnerable molecule against various mutagens including ultraviolet, toxins, chemical agent, and free radicals [10].

Recently, high-throughput sequencing platforms have been applied as powerful tools in order to investigate the association between a massive number of genetic variants and drug response [11][12].

It is shown that TAM has a mutagenic effect on the endometrium cells and increases the incidence of endometrial tumors [13].

Results of evaluating the rat hepatic tissue showed that activated TAM could bind to guanine N2-position of DNA and consequently, produce pro-mutagenic lesion [14]. More importantly, it was found that TAM mutagenicity effect induced DNA damages in human endometrial cells [15].

Emons et al. (2020) showed that TAM may have a key role in tumor progression.

It may increase the risk of uterus cancers, such as endometrial cancer and uterine sarcoma [16].

In vitro conditions, TAM would lead to the gene mutations and increased incidence of abnormal chromosomal structures in rat liver tissues [17].

All of the above-mentioned literature reviews indicated that TAM could play a critical role in the alterations of genetic variants background.

Furthermore, vaginal dryness, sleep problems, weight gain, hot flashes, and depression were reported as common TAM side effects [18].

There are several examples regarding the role of genetic variants in drug response. To achieve a therapeutic effect, there has to be an interaction between the drug and its target.

DNA variations can both increase and decrease a drugs binding affinity to its target.

As an example, genetic variations can change the antagonist role of drug into an agonist one; therefore, the most common problem of treatment procedures is resistant mutations in drug targets.

TAM blocks estrogen receptor (ER-positive cancer) in the breast cancer treatment procedure and consequently, decreases the risk of cancer recurrence.

It is an anti-estrogen hormone that inhibits the Estrogen receptors; however, its efficiency would be decreased as a result of mutations in estrogen receptors and leads to the conversion of ER-positive into progesterone-positive cancer (PR-positive cancer).

Consequently, it causes the drug resistance development and nonresponse to treatment [19].

It is noteworthy that genetic variations may contribute to drug metabolism and influence the drug response. For instance, if the drug is rapidly metabolized, its concentration will decrease due to the weaker drug action or side effects.

Considering slower metabolism procedures, higher drug levels would result in the stronger or longer actions and side effects [20].

Current study investigates the effect of TAM consumption on genetic variants background in the breast cancer cell line (MCF7).

There may be an association between genetic variants alterations and TAM treatment due to the fact that TAM is a mutagenic factor; therefore, it can affect the treatment process.

Also, it may provide a new insight to the increase of the chance of survival, decrease the side effects, and select an appropriate strategy for the therapy period.

## Materials and Methods

**Table:** Table1. More details of RNA-seq Datasets to Discover the Differential Genetic Variants.

Accession numbers of experiments	Control samples	Treated samples	Drug type (dosage)	Cell line	Duration of treatment (hr)
E-MTAB-822	1	2	TAM (1μM)	MCF7	12
E-GEOD-59536	1	1	4-OHT (1μM)	MCF7	24
E-GEOD-62613	1	1	4-OHT(1μM)	MCF7	24
E-GEOD-78199	6	6	TAM (100 nM)	MCF7	24
Total	9	10	----	----	

**hr:** hours; **TAM:** Tamoxifen; **4-OHT:** 4-hydroxy tamoxifen.

**Figure-1:**
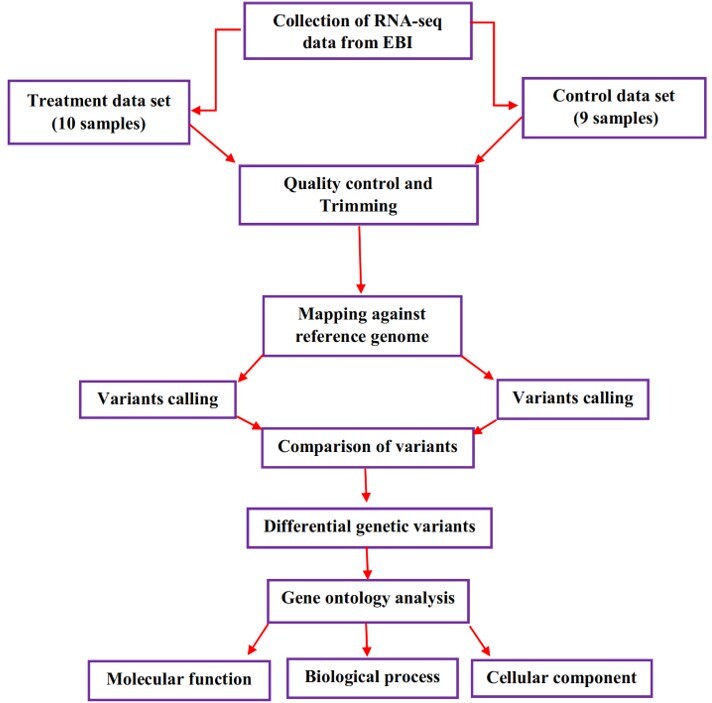
Figure-1. The summary of differential genetic variants analysis between treated breast cancer cell line (MCF7) by TAM and control group (untreated MCF7).

### 1. Data Collection

In current study, the 19 whole-transcriptome (RNA-seq) datasets of four investigations were derived from European Bioinformatics Institute (EBI) (https://www.ebi.ac.uk/). The treatment group includes 10 MCF7 cell lines treated by TAM and 4-hydroxytamoxifen (4-OHT), as well as 9 untreated MCF7 cell lines considered as the control groups. More details of collected samples were provided in Table-1. The summary of genetic variants analyses for collected samples are showed in Figure-1.

### 2.Quality Control and Trimming

Quality control functions of CLC Genomic Workbench (12) including length distribution, GC content, ambiguous base content, Phred score, nucleotide contribution, and duplicate sequences were applied in order to achieve an appropriate quality control for the collected data [21].

Due to the fact that adaptor sequences were cleaned in the achieved datasets, the adaptor trimming was not formed.

### 3.Genetic Variants Analysis

#### 3.1. Reference Genome and Alignments Analysis

The reference genome (hg38) and all annotations were downloaded from Ensembl database (www.ensembl.org). Mapping short reads against the reference genome was carried out through CLC Genomics Workbench 12 based on the following parameters: masking track=mRNA sequence, mismatch cost =2, cost of insertions and deletions=linear gap cost, insertion cost=3, deletion cost=3, length fraction=0.7, and similarity fraction=0.8 [22].

### 3.2. Variant Calling and Statistical Analysis

CLC genomics workbench 12 was applied to variant detections; also, there was no constant ploidy level in cancer cell lines. Therefore, the variant calling procedure was carried out using the low frequency algorithm on the basis of the following parameters: required variant probability (%)=95.0 ignore broken pairs=yes, minimum coverage=10, minimum count=2, minimum frequency (%)=30, base quality filter=Yes, neighborhood radius=15, minimum central quality=30, and minimum neighborhood quality=25 [23]. Chi-square test was performed with the purpose of explaining the differences of genetic variants distribution between control and treated samples.

### 3.3. Comparing the variants and gene ontology (GO) enrichment analysis

After performing the variants calling process, genetic variants of TAM-treated samples were compared with the reads of control samples in order to remove the common genetic variants between treated and control samples. The file of gene ontology association, which included the gene names and associated gene ontology terms, was downloaded from the gene ontology consortium (http://geneontology.org/) and imported to CLC Genomic Workbench 12. Moreover, differential genetic variants were applied to perform GO enrichment analysis at the levels of biological process, molecular function, and cellular component. The significance of the level of GO analysis was determined to be 0.01.

## Results

**Table:** Table2. The Mapping Summary of Short Reads against the Reference Genome.

Accession number	Samples	Total reads	Mapped reads%
E-GEOD-59536	T1	89713168	68.80
E-GEOD-62613	T2	112247072	85.91
E-GEOD-78199	T3	34981408	81.22
	T4	36012214	81.20
	T5	40160428	82.10
	T6	41384146	82.05
	T7	39870210	81.72
	T8	41063128	81.74
E-MTAB-822	T9	10069398	87.85
	T10	12018685	83.10
E-GEOD-59536	C1	97511228	66.10
E-GEOD-62613	C2	103822108	87.37
E-GEOD-78199	C3	39172180	82.47
	C4	40347538	82.44
	C5	44328838	80.16
	C6	45695050	80.15
	C7	36382948	82.53
	C8	37484422	82.50
E-MTAB-822	C9	8569125	89.25

**T:** treated samples; **C:** control samples.

**Table:** Table3. Results of Statistical Analysis of Genetic Variants Distribution between Control and Treatment Samples.

Genomic variants	P-value
SNV ^***^	0.00006
MNV ^***^	0.0001
Insertion ^***^	0.0003
Deletion ^***^	0.0012
Replacement ^***^	0.001

**SNV:** single nucleotide variations, **MNV:** multi nucleotide variations

**Table:** Table4. The Classification of Differential Genetic Variants between Control and Treated Samples

Genetic variants	Differential variants	Coding region	Non-coding regions	Amino acid changes
SNV	45	15	30	9
MNV	7	0	7	0
Insertion	5	0	5	0
Deletion	10	1	9	1
Replacement	0	0	0	0
Total	67	16	51	10

**SNV:** single nucleotide variations; **MNV:** multi nucleotide variations

**Figure-2:**
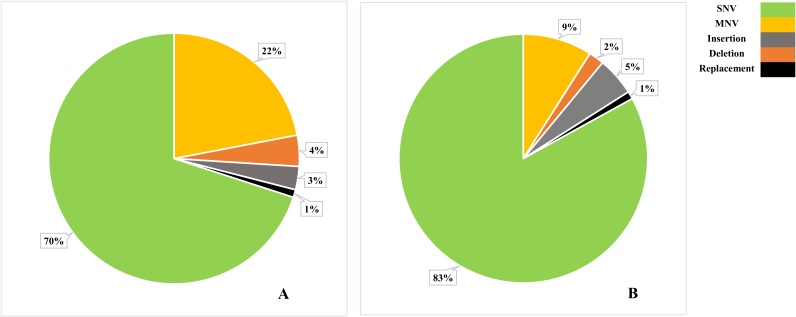
Figure-2. Frequencies of reported genetic variants in the control and treated samples. Parts A and B illustrate the frequencies of detected genetic variants within control and treated samples. A total number of 2,853,482, and 2,988,729 genetic variants were reported for control and treated samples.

### Genetic Variants Detection

Results of quality control indicated that there was not any necessary special trimming strategy for RNA-seq datasets. The average of quality control factors (per read) for studied samples were reported as the following parameters, length distribution=131.5 bp, GC content=52.35%, ambiguous base content=0.2%, Phred score=18.12, nucleotide contribution=0.5% and duplicate sequences=2.10%. However, trimming was carried out according to the Phred score and the nucleotide contribution to minimize the mapping errors.

At least ten primary bases were trimmed from 3′ side of short reads and 5% of reads that contained the lowest Phred scores were also ignored. Results of alignments of short reads against reference genome (hg 38) are provided in Table-2. Furthermore, 66%-89% was reported for the mapping percentage.

There were almost 5.8 million genetic variants identified in current study, including the single nucleotide variations (SNVs), multi nucleotide variations (MNVs), insertion, deletion, and replacement. The highest and lowest frequencies among detected genetic variants were respectively related to SNVs and replacement.

More details of genetic variants frequencies are provided in Figure-2.

To investigate the effect of TAM on genetic variants distribution within control and treated samples, a statistical analysis was separately carried out for each genetic variant on the basis of chi-square test for total genetic variants in the control and treated samples. Results showed that the genetic variants distribution between control and treated samples was significant (P≤0.05, Table-3), which indicated the possible effects of TAM on the genetic variants frequency.

Results of the comparison between genetic variants of control and treated samples indicated that there were 67 differential genetic variants. Among all of the differential variants, 16 genetic variants were located in the coding regions and 10 variants led to the change of amino acid sequence within the protein structure. Table-4 shows more details of differential genetic variants.

The process of gene ontology enrichment analysis of differential genetic variants was carried out at three levels of biological process, cellular component, and molecular function; therefore, a total number of 77 significant GO terms was reported. At the biological process level, the most repetitive of reported overlapping gene names were GEN1, HSPA5, NSMCE2, AURKA, and DDX11 candidate genes.

Results achieved from molecular function analysis indicated that the most frequent enriched candidate genes in significant GO term were IL6ST, COX15, and FNTA.

The cellular component analysis showed that nucleus and nucleoplasm were the most important cellular parts that may contribute to the hormone therapy.

## Discussion

Breast cancer is a heterogeneous disease, which is classified into three groups of ER-positive, PR-positive and Triple-Negative Breast Cancer (TNBC). Hormone therapy may be used for ER and PR positive tumors; however, TNBC could not respond to common hormone therapy [24]. TAM is a type of hormonal therapy implemented with the purpose of treating the ER-positive breast cancer; also, it can decrease the risk of invasive cancer development.

Our hypothesis regarding the role of TAM in treatment process was not approved appropriately. It was found that most of the candidate genes with differential genetic variants had dual roles as oncogenes or tumor suppressors; moreover, their exact contribution in breast cancer has not been investigated precisely.

For example, findings of the genetic variant analysis revealed that differential genetic variants between control and treated samples (under TAM therapy) were overlapped with GEN1, HSPA5, NSMCE2, AURKA, and DDX11. GEN1 (Flap endonuclease GEN homolog 1) encoded a member of Rad2/xeroderma pigmentosum group G nuclease family. As it was observed for BRCA1 and BRCA2, GEN1 contributed to resolve the Holliday junction in the homologous recombination. It is noteworthy that the Holliday junction can play a vital role in the cancer chemo-sensitivity [25]. Somatic truncating GEN1 mutations have been reported in breast cancers; therefore, it would indicate the fact that GEN1 may be a predisposition gene in breast cancer. However, it was shown that although it plays a critical role in the double-strand DNA break repair, GEN1 would not make any appreciable contribution to breast cancer susceptibility through acting as a high- or intermediate-penetrance breast cancer predisposition gene, such as BRCA1, BRCA2, CHEK2, ATM, BRIP1, and PALB2 [26].

Sun et al. (2014) suggested that GEN1 would play a vital role in DNA damage response; therefore, its alteration could lead to the breast cancer [27]. HSPA5 (Heat-shock protein 5) is considered as a marker of poor prognosis in breast cancer patients, which plays a critical role in promoting the drug resistance and metastasis [28]. A close association was observed between the cancer behaviors of heat shock proteins (HSP) family; however, all members of HSP family have not been studied completely [29].

NSMCE2 is an E3 SUMO ligase and a subunit of SMC5/6 complex that could be associated with DNA repair [30]. Although SMC5/6 complex functions were not described precisely, reports indicated that it could act as a tumor suppressor in mice [31].

AURKA (Aurora Kinase A) is a serine/threonine kinase that contributes to the regulation of cell cycle progression; therefore, it could be a potential cancer susceptibility gene [32]. Furthermore, it is considered as a promising target in the treatment processes of patients with cancer [33].

DDX11 is a DNA helicase that plays a role in DNA replication, sister chromatid cohesion establishment, and general chromosome structure. The effects of DNA helicases among patients with cancer are dependent upon their genetic background and tumor type; however, it has not been illustrated precisely and there are various reports of their activities. For example, it was suggested that DNA helicase may have a tumor suppressor function, and the expression level of several DNA helicases at pre-cancerous stages would be increased significantly [34].

At the molecular function level, results of GO analysis indicated that differential genetic variants were associated with FNTA, IL-6, and COX15 candidate genes.

FNTA is located on chromosome 8 and encodes the subunit alpha of protein farnesyltransferase (FTase) enzyme (UniProtKB: P49354).

It was found that FNTA could be a key gene for tumor progression; moreover, its abnormal copy numbers were associated with pathological transformations of breast cancer. Therefore, it could be considered as a main target of developing drugs [35]. Interleukin-6 (IL-6) as a cytokine released by various cells such as cancerous cells contributed to the expansion and differentiation of tumor cells [36].

It was also shown that IL6ST may respectively act as a main factor and a tumor suppressor gene in TNBC progression, and diagnosis and treatment procedures [37].

Additionally, IL6ST candidate gene was reported as a specific candidate gene for TNBC [38]. COX15 gene encodes cytochrome C Oxidase subunit 15 and contributes to mitochondrial respiratory chain (UniProtKB: Q7KZN9). Gao et al. (2017) reported that the high-level expression of COX5B gene was associated with a poor prognosis in breast cancer [39].

It was suggested that the level of COX5B protein may be related to the tumor size; also, its up-regulated form showed a worse disease free-survival. However, there was not enough evidence to illustrate the clinical implications of COX5B in breast cancer.

### Limitations

In this study, we did not generate the RNA-seq datasets and they were downloaded from different experiments. It is difficult to find datasets with the same condition. However, we tried to select studies that performed in the same conditions. But there are differences between studies. For example, the dosage of TAM was not the same in all studies. In addition, the RNA sequencing platforms were different between experiments. It should be noted that it can affect the results. Clearly, by generating the RNA-seq datasets in the similar experimental conditions the reported results will be more reliable.

## Conclusion

Results of differential genetic variants analysis between control and treated samples indicated that the most reported candidate genes had dual roles as oncogenes or tumor suppressors. Therefore, it was suggested that TAM could not have any significant role in an effective treatment through changing the genetic variants background.

## Conflict of Interest

The authors declare no conflict of interest.
